# Suboptimal Micronutrient Intake among Children in Europe

**DOI:** 10.3390/nu7053524

**Published:** 2015-05-13

**Authors:** Boris Kaganov, Margherita Caroli, Artur Mazur, Atul Singhal, Andrea Vania

**Affiliations:** 1 Nutrition and Health Clinic, Moscow 109012, Russia; 2 Nutrition Unit, Department of Prevention, Azienda Sanitaria Locale Brindisi, Brindisi 72021, Italy; E-Mail: margheritacaroli53@gmail.com; 3 Department of Pediatrics, University of Rzeszow, Rzeszow 35-350, Poland; E-Mail: drmazur@poczta.onet.pl; 4 Childhood Nutrition Research Centre, Institute of Child Health, London, WC1N 1EH, UK; E-Mail: a.singhal@ucl.ac.uk; 5 Centre of Dietetics and Nutrition, Sapienza University, Rome 00161, Italy; E-Mail: andrea.vania57@gmail.com

**Keywords:** pediatric, nutrition, micronutrient, deficiency, Europe, diet, supplements, public health

## Abstract

Adequate dietary intake of micronutrients is not necessarily achieved even in resource-rich areas of the world wherein overeating is a public health concern. In Europe, population-based data suggests substantial variability in micronutrient intake among children. Two independent surveys of micronutrient consumption among European children were evaluated. Stratified by age, the data regarding micronutrient intake were evaluated in the context of daily requirements, which are typically estimated in the absence of reliable absolute values derived from prospective studies. The proportion of children living in Europe whose intake of at least some vitamins and trace elements are at or below the estimated average requirements is substantial. The most common deficiencies across age groups included vitamin D, vitamin E, and iodine. Specific deficiencies were not uniform across countries or by age or gender.  Micronutrient intake appears to be more strongly influenced by factors other than access to food. Substantial portions of European children may be at risk of reversible health risks from inadequate intake of micronutrients. Despite the growing health threat posed by excess intake of calories, adequate exposure to vitamins, trace elements, and other micronutrients may deserve attention in public health initiatives to optimize growth and development in the European pediatric population.

## 1. Introduction

While recognizing that comprehensive data regarding diet and nutrition among children in Europe remain limited, well-documented nutritional deficiencies do exist across the European pediatric population. The aim of this review article is to raise awareness of the importance of ensuring children who have ready access to food are maintaining adequate levels of micronutrients through proper food choices and appropriate use of nutritional supplements.

Based on the clear relationship between adequate intake of vitamins as well as other micronutrients and health, authorities in most countries of Europe publish recommendations for appropriate population-based daily nutrient intake [1]. In 2006 the European Food Safety Authority (EFSA) published its first consensus document regarding appropriate consumption within the European Union [2]. In that document, appropriate levels were defined as dietary reference values (DRVs). These and other terms, like dietary reference intakes (DRIs), recommended dietary allowances (RDAs) and estimated daily requirements (EDRs), express best estimates of nutrient intakes that will reduce the risk of adverse health consequences associated with a specific nutrient. Table 1.

**Table 1:** Common Terms for Describing Nutritional Values.

DRV (*Dietary Reference Value*) or EAR (*Estimated Average Requirment*): how much of a nutrient meets the needs of 50% of the healthy subjects of a specific population’s group
**DRI** or **RDA** (*Dietary Reference Intake* or *Recommended Dietary Allowance*): how much of a nutrient meets the needs of 97.5% (mean + 2SD) of healthy subjects of a specific population group
**EDR** (*Estimated Daily Requirement*): estimate of nutrient intakes that will reduce risk of adverse health consequences
**AI** (*Adequate Intake*): how much of a nutrient is adequate for a population’s group, according to the average intakes of apparently healthy people
**UL** (tolerable *Upper* intake *Levels*): maximum intake of a nutrient not bound to any adverse effect
**LTI** (*Lowest Threshold Intake*): lowest acceptable intake, under which nearly all individuals in a population’s group will not maintain metabolic integrity and efficiency

In the absence of reliable information of an absolute threshold at which a nutrient deficit leads to complications, DRVs are often derived from average intakes in a healthy population. Values are typically set at two standard deviations above the average to accommodate variability in physiologic demands [3]. Discrepancies between DRVs from different guidelines can arise from several causes including differences in the sampled populations and in the methods with which studies were conducted. Nevertheless, comparisons of population-based nutritional recommendations, such as those included on food labels, typically reveal relatively modest differences across Europe and countries outside of Europe [4].

In children, the disparity among available nutritional guidelines has been modest within Europe where many countries continue to issue independent recommendations for the pediatric population. The differences stem from methodology regarding how data is collected and interpreted across age ranges that are not necessarily stratified identically. For example, in Italy recommendations are made for five age groups in post-breastfed children (ages 1–3, 4–6, 7–10, 11–14, 15–17 years), whereas a recently published Nordic (Denmark, Finland, Norway, and Sweden) consensus document divided infants aged younger than 2 years into three groups and older children into four groups (ages 2–5, 6–9, 10–13, 14–17 years) [5] Recently published EFSA guidelines were limited to infants and children of 36 months of age or younger with multiple stratifications within this age range [6]. 

Specific stratifications are relevant to nutritional analyses. In general, children require higher levels of many nutrients proportional to body weight than adults [7] but their specific needs evolve rapidly at different points in growth. Metabolically active organs, such as the brain, liver, and heart, represent a far greater proportion of body weight in infants than adults and have reached 50% of adult size by 2 years of age [8]. In deriving data about health nutrient intake from a sample population, the specific age stratifications could therefore influence normative data. Moreover, the methodologies for evaluating food consumption, such as 7-day dietary recall or standardized questionnaire, as well as the statistical methods for data analysis have the potential for producing differences that complicate comparisons.

Finally, the presentation of data from different nutritional guidelines may vary. In the EFSA guidelines, tables outlining nutrients represent a synthesis of available data in a format intended to be simple to consult. In contrast, tables in the Italian guidelines include information on the quality of data, drawing attention to values for which average intake (AI) has been substituted for average requirements (AR) when reliable data to calculate ARs are absent. This increased detail may be of use when considering nutrition in special populations, such as those with gastrointestinal (GI) dysfunction, even if it renders the tables more complex.

Due to potentially irreversible physical or cognitive deficits, the consequences of micronutrient deficiency are potentially greater in children than adults. Many of these deficiencies and complications, such as inadequate vitamin D intake leading to abnormal bone formation [9], are well known and readily recognizable. Others, such as the impact of micronutrient deficiency on cognition, may be both complex and subtle [10]. Micronutrients implicated in cognitive development include iodine [11], for which deficiencies have correlated with lower intelligence quotient (IQ) scores [12], iron, which is important for oxygen transport to the brain [13], as well as zinc [14] and thiamine [15].

In Europe, deficiencies in micronutrients are mostly related to the quality of the diet but not to the quantity of food consumed. For this reason, the risks of micronutrient deficiency have the potential to persist in otherwise resource-rich areas of the world. Due to the importance of these micronutrients for growth and development, risks posed by deficiencies may be greater in children, who may also have lower stores of micronutrients than adults to bridge periods of deficiency. In both, nutritional assessments have the potential to avoid preventable disease.

## 2. Methods

Two studies, employing different methodologies, have evaluated micronutrient consumption among children living in Europe. In a report from the Directorate General of the European Commission, Health and Consumers, nutritional data was compared across regions of Europe [16]. In this study, data was collected utilizing the EU-supported Data Food Networking (DAFNE) methodology over a one year period in order to capture seasonal variations in food intake. In the other study, representative dietary survey data were collected and compared from Belgium, Denmark, France, Germany, The Netherlands, Poland, Spain, and the United Kingdom (UK) [17]. In this study, a number of different design and dietary assessment methods were used in the surveys, including single or repeated 24 h recalls; 2, 3, 4 or 7 day prospective food records; estimated or weighed amounts consumed; and modified dietary histories with a reference period of 4 weeks. All the surveys, except for those from Belgium and Poland, covered all seasons of the year.

In both studies, data were stratified by types of micronutrients within predefined age groups. Although the methods of data collection and analysis varied, including definitions of adequate and inadequate micronutrient consumption, these two sets of data provide a basis for considering potential trends. They provide a context for evaluating the variability in diet and its potential impact on endpoints relevant to pediatric health.

No human subjects nor their data were prospectively collected in the execution of this work. All of the data used herein were extracted from published sources.

## 3. Results

In the European survey, nutritional information from 16 countries was collected for analysis in four geographical regions. The north region included countries from Scandinavia. The east region included Germany, Austria, and countries of Eastern Europe. The south region included Greece, Italy, and the Iberian countries. The west region included Belgium, France, Ireland, The Netherlands, and the UK. Reference values for adequate nutrient intake were drawn from 2003 World Health Organization (WHO) guidelines [18].

The data were not collected concurrently but in country-specific surveys conducted between the years 2000 and 2008. There were also differences in the methodology of collection, such as 3-day *vs*. 7-day dietary recall. Despite efforts to homogenize the data, the authors emphasized its limitations. The findings were considered suitable to an overview of nutrition in Europe, which was the objective of this evaluation, but of limited reliability for rigorous conclusions about relative nutritional intake for cross-country comparisons.

**Figure 1:**
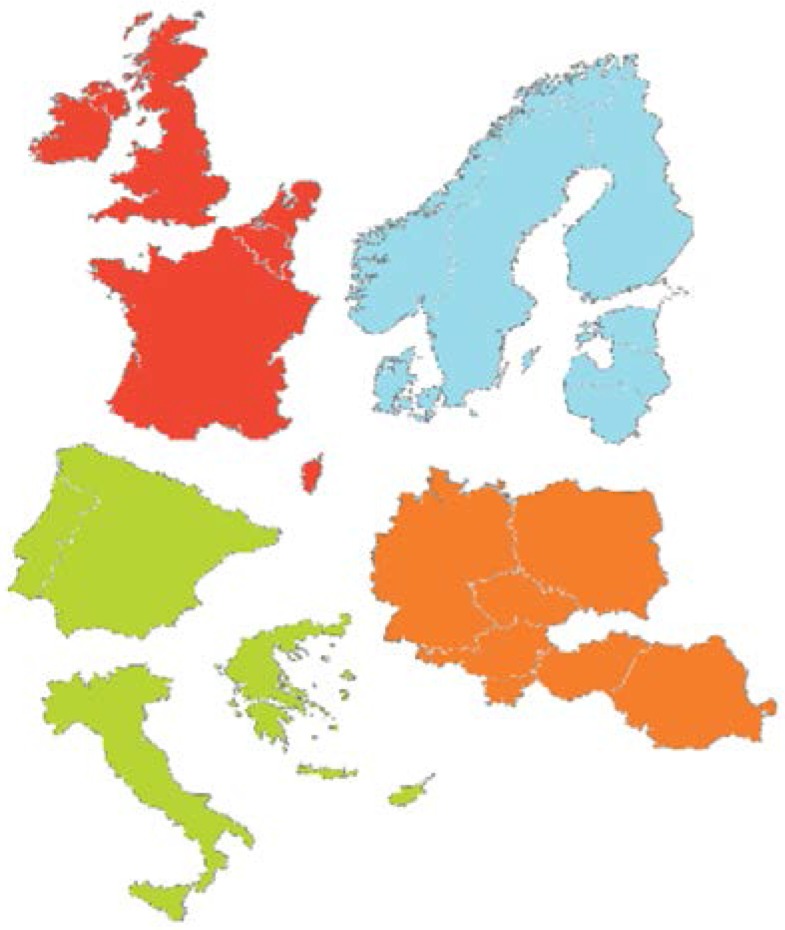
Folate and Vitamin D levels that fell below recommended reference values compared to normal ranges of Vitamin B6 levels in children in different regions of Europe.

Across the four age groups evaluated (4 to 6 years, 7 to 9 years, 10 to 14 years, and 15 to 18 years) the most commonly observed deficiencies involved vitamin D, folate, iron, calcium, iodine, and phosphate. For most of the remaining micronutrients, such as the trace minerals selenium and magnesium, and the vitamins B6 and vitamin C, deficiencies were rare or not observed. However, there was substantial regional variability in reported deficiencies by individual nutrient within age groups and gender (Figure 1).

The most consistent finding across all age groups was deficiency in vitamin D and folate. Both the minimum and maximum average intake of vitamin D and folate fell below reference standards in most age groups in all four geographic regions. Although both the minimum and maximum average intake are relevant to epidemiologic studies of nutrition, the terms differ for their relevance to the definition of malnutrition. An intake below the minimum average, unlike the maximum intake, indicates the potential for inadequacy, or malnutrition as it applies to that nutrient.

**Table:** 

Age (years)	Region	Sex	Folate (Ref 300 mcg)	Vitamin D (Ref: 5 mcg)	Vitamin B6 (Ref: 0.7 mg)
**4–6**	**North**	Male	135–256	2.3–6.8	1.1–1.6
		Female	132–235	2.0–6.5	1.0–1.5
**7–9**	**North**	Male	204–290	2.5–6.4	1.3–2.5
		Female	187–264	2.2–5.1	1.2–1.6
**4–6**	**South**	Male	198	2.3	1.6
		Female	199	2.2	1.6
**7–9**	**South**	Male	242	2.8	1.8
		Female	211	2.1	1.7
**4–6**	**Central-East**	Male	190–214	1.8–2.3	1.5–1.8
		Female	164–190	1.5–2.3	1.2–1.9
**7–9**	**Central-East**	Male	154–229	1.5–2.8	1.2–1.8
		Female	145–212	1.5–2.7	1.1–1.8
**4–6**	**West**	Male	120–225	2.2–2.4	1.3–1.8
		Female	109–196	1.9	1.2–1.7
**7–9**	**West**	Male	144–256	2.2–2.9	1.3–2.2
		Female	133–226	2.4–2.8	1.2–1.9

**Figure 2:**
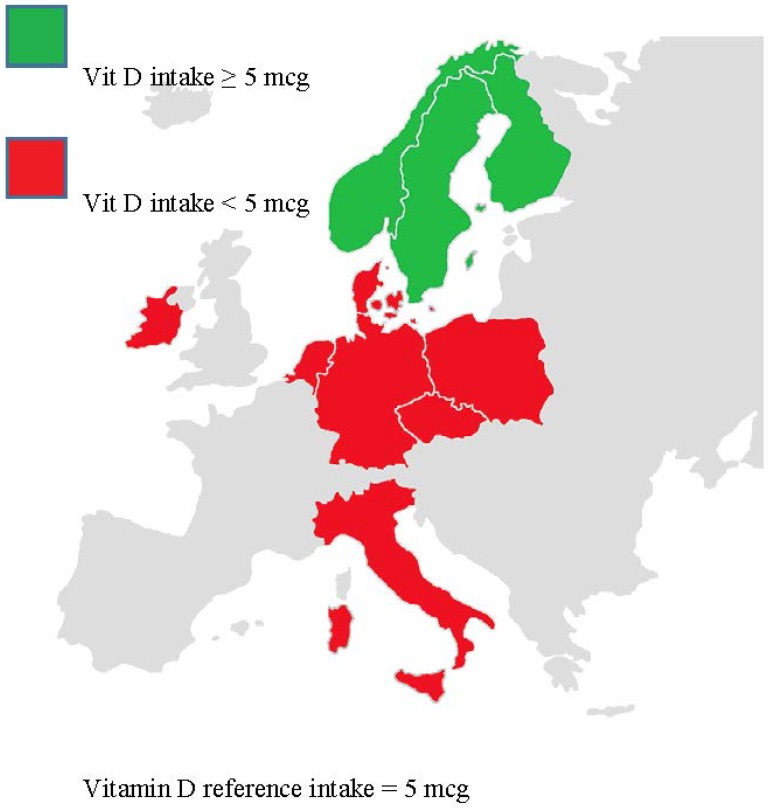
Vitamin D intake in European children aged 4–18 years old.

No deficiencies other than vitamin D and folate were observed as consistently (Figure 2), but other types of deficiencies were observed frequently. For example, the maximum average intake of iron was below recommended levels among females aged 4–6 years in the north and west and in all older females from any region. The maximum average intake of iodine was below recommended levels in the central region for both sexes at all age groups and for older females in the west region.

Among children aged 7–9 years, both the minimum and maximum average intake of iron also fell below the reference standard among females in the north, south, and west as did iodine for females in the east. Minimum averages, but not maximum averages, were below reference standards for calcium among both males and females in the north and east, and, for iron, among males and females in the north and west. For all other nutrients, both minimum and maximum average intakes exceeded reference standards.

Relative to younger children, the frequency with which both the minimum and maximum average intake for any given nutrient fell below the reference standard was greater in both those aged 10 to 14 years and in adolescents. In those aged 10–14 years, this included calcium among males and females in all four regions and iron among males and females in the north, south, and west. Both minimum and maximum average intake of iron was also below reference standards among both girls and boys in all regions with the exception of males in the east. Maximum and minimum average iodine intake was below the reference standard for both genders in the east and west. In the oldest age group, both the minimum and maximum average intake of calcium fell below the reference standard in both genders in most regions and average minimum intake were commonly deficient for iron, zinc, and iodine.

In the second study, which included both adults and children, nutritional experts from European countries were invited to submit data that met predefined quality criteria. Data from eight countries were included. The nutrients selected for analysis included those for which there were reference values and for which there were known or suspected deficiencies. These were calcium, copper, iodine, iron, magnesium, potassium, selenium, zinc, and the vitamins A, B1, B2, B6, B12, C, D, E, and folate. The reference values were taken from values originally established in the UK in 1991 [19], or for those missing, from the 2004 Nordic Nutrient recommendations [20]. The age of these reference values in the context of dietary changes is a potential weakness of this study but more recent UK values were unavailable. Results were expressed in several ways, including the proportion of the population with an estimated daily intake below the lower reference nutrient intake (LRNI) and estimated average requirement (EAR).

For the majority of nutrients evaluated, the proportion of children with daily intakes below the EAR, suggesting a potential for a health risk was measurable in at least some countries in one or more of the five pediatric age ranges evaluated (ages 1–3 years, 4–6 years, 7–10 years, 11–14 years, 15–17 years) (Table 2). Iodine is one example. For this nutrient, 61% of girls and 55% of boys aged 4–10 years in Germany had intakes below the EAR. Iron is another example. In this case, 94% of girls in Germany aged 4–10 years had average intakes below the EAR. The average intakes of vitamin D, a third example, were below the EAR in essentially all children in every age group and country.

**Table 2:** Percent of children with intakes of select micronutrients intake estimated average requirement (EAR) as reported in various surveys in several European countries.

Country	Iodine		Iron			Magnesium	Selenium	Zinc	
	C ♂ (75–82.5 μg/day)	C ♀ (75-82.5 μg/day)	T ♂♀ (5.3 mg/day)	C ♀ (4.7–6.7 mg/day)	A ♀ (8.7 mg/day)	A ♀ (230–250 mg/day)	A ♀ (33.8–45 mg/day)	C ♀ (5.0–5.4 mg/day)	A ♀ (5.5–7.0 mg/day)
BE	-	-	23.4	10.2	-	0.4	-	7.6	-
DK	0.0	0.0		18.8	93.9	0.5	79.6	4.9	23.0
FR	11.8	25.3		3.5	73.6	4.7	54.1	4.6	18.4
DE *	55.1	60.8		5.5	24.8	1.6	-	13.8	6.0
PL	41.9	45.5	55.1	28.3	67.5	11.2	-	27.0	19.2
ES	-	-		0.3	23.8	0.3	-	-	-
NL	-	-	32.0	12.0	85.1	0.2 ^†^; 5.6 ^‡^	67.9	39.0; 13.0	21.0
UK	9.2	16.3	32.3	25.9	67.0	8.6	60.8	27.1	47.5

Reference values for respective nutrient in (parenthesis); * Age range for a child in Germany is 6–10 years old; T = Toddler (<3 years old); C = Child (ranges between 4–10 years old depending on country); A = Adolescent (11–17 years old); ^†^ Children 4–6 years old; ^‡^ Children 7–10 years old.

Even when deficiencies were uncommon across most countries, there were exceptions. For example, the proportion of patients with an average intake of vitamin E below the EAR was consistently low across all countries with the exception of boys aged 4–10 in Belgium, where more than 20% were deficient by the reference standard. In addition, it is reasonable to consider whether low prevalence rates in relative terms are still clinically meaningful. In children who face complications for readily reversible nutrient deficiencies, small but measurable prevalence rates may signal a need for simple screening so that dietary modification or supplementation can be implemented.

## 4. Discussion

The obstacles to determine adequate micronutrient intake are complex. In regions where fruits, fresh vegetables, and proteins are readily available and well represented in the average diet, nutritional deficiencies are likely to be uncommon. Yet, dietary decisions made by individuals within such regions may still lead to nutritional deficiencies, including nutritional deficiencies concurrent with excess body weight. Despite modern food distribution, which has improved consistent access to diverse food groups in countries with short growing seasons, food choices by parents for young children and older children for themselves are not necessarily influenced the goal of healthy eating.

Public health initiatives in Europe to address common nutritional deficiencies vary by country. For example, iodine fortification of salt is mandatory in some countries such as Denmark but voluntary in others, such as Finland and Italy. Fortification of milk with vitamin D is required or strongly encouraged in most countries but fortification of milk products or margarine with vitamin A or E is less common. Fortification of fruit juices with vitamin C is not mandatory and may vary by country according to consumer demand.

Differences in fortification policies are likely to explain some portion of the disparity in the prevalence of some nutrient deficiencies among children living in Europe but the diet of any individual child may deviate grossly from those of peers. Parents who avoid individual food groups due to preference or belief, or children who refuse specific food categories, are vulnerable to deficiencies even when consuming an otherwise quantitatively well balanced diet. Indeed, the “picky eater” child is a well-recognized phenomenon with prevalence rates estimated as high as 50% in children 24-months-of age [21] A greater relative risk of nutritional deficiencies is likely in children on exclusion diets, such as those on a vegetarian (especially vegan), gluten-free, or lactose-free diet.

Due to the evidence of persistent deficiencies among children, a systematic approach to ensuring adequate access to vital vitamins, trace elements, and other nutrients essential to healthy growth, such as calcium, is reasonable in routine pediatric care. In otherwise healthy children, a formal dietary assessment, although potentially valuable for increasing the rate of detection, may not be justified by time and expense, but general questions about diet may identify potential inadequacies. Several relatively simple tools, such as the Child and Diet Evaluation Tool (CADET) [22], have been validated for identifying children with low intake of fruits and vegetable. Clinical inquiries about diet have the potential to reveal inadequacies at the same time that they serve to convey the message that a healthy diet plays a role in disease prevention.

When informal dietary surveys suggest potential problems in nutrient acquisition, further evaluation, including a referral to a nutritionist or dietician, may be appropriate. It is important not to underestimate the complexity of adequate nutrition. The interplay and heterogeneity of variables that affect how micronutrients are metabolized and stored remains a focus of study. In experimental studies, for example, metabolism of iron is affected by dietary exposure to both zinc and nicotinic acid [23] Clinical studies in resource-poor areas of the world have suggested that coexisting micronutrient deficiencies can impair the response to single micronutrient replacement therapy [24].

In children suspected or at risk of micronutrient deficiencies, nutritional supplementation may be a valuable approach for ensuring adequate intakes. Supplementation of specific nutrients is helpful in those with chronic diseases affecting liver or gut function, such as Crohn’s disease, or in those on exclusion diets liable to limit intake of specific vitamins. There is also a strong rationale for supplements in preventing deficiencies in otherwise healthy children at risk for micronutrient deficiencies. This may be particularly important in specific stages of development, such as vitamin D in infants or vitamin D and iron in toddlers and other young children in whom there is evidence of adverse health consequences when these nutrients are deficient. Published studies have associated supplementation of vitamin D, which appears to enhance metabolism in several organs other than bone, such as immune function [25], with improved nutrient adequacy in both children and adults. [26,27] This finding has lead public health agencies in some countries (e.g., the U.K.) to recommend supplementation, particularly for children younger than age 5 years.

Due to the evidence that there may be interactions within a healthy diet important to the metabolism of nutrients, supplementation should not be considered a substitute for a well-balanced healthy diet. Although suitable for adjunctive use when nutrient deficiencies are known or suspected, nutritional supplementation does not provide any additional benefit in individuals who are already obtaining adequate nutrients by diet alone. Specific supplements should be recommended on the basis of the nutrients relevant to the risk of deficiency at recommended dosages. Whether or not supplements are being considered, parents and older children should receive explicit information on the essential role of a healthy diet to maximize growth and development while minimizing health risks.

In Europe, the persistence of nutritional deficiencies in the midst of an obesity epidemic should not be misunderstood as a paradox. Ready access to food does not ensure healthy food choices required to achieve adequate nutrition. In young children, food choices may be dictated by adults responding to well marketed prepared products high in fat and low in nutritional value. Modern food distribution has largely eliminated seasonal gaps in adequate access to fruits and vegetables but individual choice can thwart efforts to promote healthy eating. The data from two large surveys suggest that prevalence rates of selected nutritional deficiencies among European children are clinically meaningful. The rates for specific nutrient deficiencies vary by country but several, including vitamin D, folate, and iron, are common. Due to the threat these deficiencies pose for impaired growth and other adverse health consequences, the data should encourage public health initiatives designed to improve nutrition on a population basis and to consider routine inquiries about diet in the care of healthy children. In children with normal metabolism, the risks posed by nutritional deficiencies may be circumvented if detected and reversed in early stages of development.
